# Lafayette at Yorktown

**Published:** 1881-12

**Authors:** 


### LAFAYETTE AT YORKTOWN.

The personal bravery of Lafayette is fitly illustrated in the following extract from a forthcoming publication by the Rev. A. B. Muzzey, of Cambridge:

"In the great struggle at Yorktown in the early part of October, 1781, the British were left masters of no external works except two large redoubts; these Washington determined to take. Lafayette, at the head of the American Light Infantry, was ordered to attack the redoubt on the left of the besieged troops. He thought nothing but a bold and rapid onset would enable young soldiers like his to carry intrenchments defended by disciplined troops. He ordered the whole of his division at the word of command to fire, and formed his men in solid column. He then headed them himself, charged sword in hand through the mound, in the face of the enemy's fire, forced his way into the redoubt, and in a few minutes carried it with the loss of only a handful of men. After a five-days' contest, in which it is said Washington fired the first gun himself, Cornwallis on the 17th of October demanded a parley, and on the 19th surrendered his army, and in presence of Generals Rochambeau and Lafayette his sword was delivered to Washington. It is a singular coincidence that in 1824, forty-three years after this battle, Lafayette came to Yorktown, and stopped at the very house then occupied by Cornwallis, and the rooms were lighted by a remnant of the wax candles once used by him."

